# The Academy of Medicine

**Published:** 1881-10

**Authors:** 


THE ACADEMY OF MEDICINE held its annual meeting September 20th, in the College of Physicians and Surgeons, at Twenty-third Street and Fourth Avenue. The meeting was called to order by its presiding officer, Dr. E. T. Caswell, of Providence, R.I. After the election of new members, Dr. C. Carrol Bombaugh read a paper on the "Prevalent misuse of the term Allopathy." The Legislative Committee appointed at the last annual meeting, consisting of Drs. H. O. Marcy and R. J. Dunglinson, made their report, in which they discussed the condition of legislation in the different States in regard to the practice of medicine. This report embraced matters of vital importance to all, and throws much light on the present status of the case. The following officers were elected for the ensuing year: Presidents, Dr. Trail Green; Vice-President, Dr. James F. Strawbridge, Louis Elsberg, and C. C. Bombaugh; Dr. R. J. Dunglingson, Secretary; Dr. C. C. Lee, Treasurer.

At the evening session, the President, Dr. E. T. Caswell, delivered an address upon "Reform in Medical Education the Aim of the American Academy." The Doctor, referring to the skill displayed by President Garfield's physicians, especially by the consulting surgeons, who are members of the Academy, said:

At a moment like the present our thoughts have a common direction. One sad sentiment prevails in every breast—the loss that our country has sustained in the death of the President of the United

States. As physicians throughout the length and breadth of the land, we have for weeks scanned with the most intense anxiety the daily reports that have been received from the sick-room. But now all our fears and hopes are buried together. The calm courage and vigorous constitution of the illustrious patient, the undoubted skill and self-sacrificing devotion of his medical advisers, have been of no avail. It is for us to place on record the high appreciation of the lofty virtues that formed his character and that had already endeared him to the whole people; and our calm conviction that our colleagues, upon whom have rested such grave responsibilities, have discharged them with unfaltering fidelity and assiduity.

At the close of the address the following resolutions were unanimously adopted:

*Resolved*, That in common with all the citizens of our land we lament with deep grief and profound sorrow the untimely death of James A. Garfield, President of the United States, whose magnificent career down to his last struggle with death was marked with such extraordinary manifestation of all that constitutes a typical manhood as to enshrine his memory forever in the hearts of his countrymen as one of the wisest, bravest and best of men.

*Resolved*, That our heartfelt sympathy is hereby tendered to the true, loving wife, whose devoted attention to her suffering husband has made her name one of the brightest among those of American women; to the noble aged mother, whose joy it was to see her son attain the highest place in the land as the reward of years of honorable and untiring exertion; and to the children who are mourning the loss of a wise and loving father. May He, who can alone bring consolation to the deeply afflicted be very near to them in their dark hours of sorrow and grief.

*Resolved*, That the prudent, faithful and indefatigable attentions of the attending physicians upon President Garfied and of our respected fellow-members, the consulting surgeons, merit the approval of their medical brethren, and that in the opinion of the fellows of the American Academy of Medicine, all seems to have been done for the illustrious patient that scientific knowledge and practical skill could suggest to ward off the fatal effects of the assassin's ball.

*Resolved*, That a copy of these resolutions be transmitted by the

Secretary to the family of President Garfield, and to each of the physicians in attendance upon his case.

The meeting then adjourned, to meet next September, in Philadelphia.

